# Association of liberal versus restrictive oxygen saturation targets with mortality in mechanically ventilated stroke patients: a multicenter retrospective propensity-matched analysis

**DOI:** 10.3389/fneur.2026.1840183

**Published:** 2026-10-19

**Authors:** Amir Ghafarian, Jawad Saad, Muaaz Wajahath, Mohammad Ghafarian, Wael Saasouh, Khaled Saleh, Mosab Nasser, Abbasali Badami, Awni D. Shahait, Andrea Loggini

**Affiliations:** 1 International University of the Health Sciences, Basseterre, Saint Kitts and Nevis; 2 FAJR Global, Houston, TX, United States; 3 Wayne State University School of Medicine, Detroit, MI, United States; 4 Michigan State University College of Human Medicine, East Lansing, MI, United States; 5 Department of Family and Community Medicine, University of Toronto, Toronto, ON, Canada; 6 Outcomes Research Consortium^®^, Houston, TX, United States; 7 Trauma and Acute Care Service, Southern Illinois Healthcare, Carbondal, IL, United States; 8 Southern Illinois University School of Medicine, Carbondale, IL, United States; 9 Brain and Spine Institute, Southern Illinois Healthcare, Carbondale, IL, United States

**Keywords:** ICU, mechanical ventilation, mortality, oxygenation, stroke, acute ischemic stroke, intracerebral hemorrhage, TriNetX

## Abstract

**Background:**

Optimal oxygen saturation targets in mechanically ventilated stroke patients remain uncertain. While restrictive oxygen strategies are increasingly adopted in general critical care, their impact in acute ischemic stroke (AIS) and intracerebral hemorrhage (ICH) are unclear. We evaluated the association between restrictive versus liberal oxygen saturation targets during the first 48 h of mechanical ventilation and short-term mortality in stroke patients.

**Methods:**

We conducted a multicenter retrospective cohort study using the TriNetX database (2006–2026). Adult patients (≥18 years) with a stroke diagnosis who required invasive mechanical ventilation and had documented peripheral oxygen saturation (SpO₂) values within the first 48 h of ventilation were included. Patients were stratified according to early oxygen saturation targets: restrictive (SpO₂ 92–96%) versus liberal (SpO₂ ≥ 97%). Propensity score matching (PSM) by demographics, comorbidities, and stroke severity was performed separately within the combined stroke cohort and prespecified AIS and ICH subgroups to balance demographic and clinical covariates. The primary outcome was all-cause mortality at 30 and 90 days. Secondary outcomes included 30-day tracheostomy and ventilator-associated pneumonia. Time-to-event analyses were performed using Kaplan–Meier methods and Cox proportional hazards models. *p*-value was set at 0.05.

**Results:**

Among 17,207 mechanically ventilated stroke patients, 2,366 received restrictive and 14,841 liberal oxygen saturation targets prior to matching. After PSM, 2,350 patients remained in each group. Restrictive oxygenation during the first 48 h was associated with higher mortality at 1 month (HR 1.13, 95% CI 1.03–1.23; *p* = 0.008) and 3 months (HR 1.11, 95% CI 1.02–1.20; *p* = 0.014). In AIS, restrictive oxygenation was similarly associated with increased mortality at 1 month (HR 1.26, 95% CI 1.11–1.43) and 3 months (HR 1.22, 95% CI 1.08–1.37). No statistically significant mortality difference was observed in ICH. Restrictive oxygenation was associated with lower 30-day tracheostomy rates (8.5% vs. 13.1%; RR 0.653, *p* < 0.001), with no difference in VAP.

**Conclusion:**

Restrictive oxygen saturation targets during the first 48 h of mechanical ventilation may be associated with higher short-term mortality in stroke, driven primarily by the AIS group. These findings suggest that early oxygenation strategy may have subtype-specific implications and warrant prospective evaluation in stroke focused randomized trials.

## Introduction

Stroke remains the leading cause of death and long-term disability worldwide (1). A subset of patients with acute ischemic stroke (AIS) or intracerebral hemorrhage (ICH) require intensive care unit (ICU) admission and mechanical ventilation due to depressed consciousness, loss of airway protection, or respiratory failure (2–4). In these critically ill patients, outcomes are determined not only by the primary cerebrovascular injury but also by secondary acquired pathologies during ICU care. The most common medical complications in ICU in stroke patients include thromboembolic complications and lung-related infective or inflammatory pathologies (5).

In mechanically ventilated patients, continuous oxygen titration is routinely implemented to maintain target peripheral oxygen saturation (SpO₂) (6, 7).

Although preventing hypoxemia is universally prioritized (1, 8), the optimal upper SpO₂ target remains uncertain (6, 7). Liberal saturation targets may be used to maximize oxygen delivery and provide perceived safety margins during periods of physiologic stress. However, exposure to higher oxygen levels has been associated with potential harms in critically ill populations, including oxidative stress and cerebrovascular effects that could theoretically worsen neurologic injury (9). Conversely, restrictive saturation targets aim to limit potential oxygen-related toxicity, but may raise concerns about insufficient oxygen delivery in the brain tissue, particularly in patients with acute cerebrovascular pathologies and brain tissue at risk of oligoemia or with survivable penumbra (6, 7).

Despite the clinical importance of SpO₂ titration in neurocritical care, evidence specific to mechanically ventilated stroke patients remains limited (10, 11). Importantly, AIS and ICH represent distinct disease processes (1, 8), and the balance between risks of hypoxemia and potential harms from higher oxygen exposure may differ between these subtypes (10–12). In practice, this uncertainty contributes to wide variation in oxygenation targets, even among patients with similar neurologic severity and respiratory support needs. Furthermore, the present literature that has investigated the association between hyperoxia and mortality in mechanically ventilated stroke patients are often limited by small sample sizes, or lack of propensity score matching of baseline severities between patient comparisons.

To address this gap, we performed a multicenter retrospective cohort study using a large federated electronic health record network to evaluate the association between restrictive versus liberal oxygen saturation ranges and mortality among mechanically ventilated stroke patients. Using propensity score matching to reduce confounding, we compared all-cause mortality over short-term follow-up and assessed secondary clinical outcomes, with prespecified subgroup analyses in AIS and ICH. We hypothesized that early oxygen saturation would be associated with short-term mortality in mechanically ventilated stroke patients, and that this association would differ between ischaemic and haemorrhagic stroke.

## Methods

### Study design and data source

TriNetx is a research network compliant with the Health Insurance Portability and Accountability Act and the US federal law which protects the privacy and security of health care data, including de-identified data as per the deidentification standard of the HIPAA (Health Insurance Portability and Accountability Act) Privacy Rule. The TriNetX database performs internal and extensive data quality assessment with every refresh based on conformance, completeness, and plausibility (13). This article follows the STROBE (Strengthening the Reporting of Observational Studies in Epidemiology) reporting guideline (Supplementary Table 1) (14).

All clinical variables were extracted from structured EHR (Electronic Health Record) fields within the TriNetX platform using standardized coding systems. Stroke diagnosis was ascertained using ICD-10-CM codes within the timeframe 2006–2026. Mechanical ventilation was identified using CPT (Current Procedural Terminology) procedure codes. Peripheral oxygen saturation was identified using LOINC code 59408-5. Comorbidities were identified using ICD-10-CM diagnostic codes documented prior to the time of the index event. SpO₂ values within TriNetX represent discrete pulse-oximetry results documented in the contributing organization’s electronic health record and transmitted to the network; the platform does not ingest continuous bedside monitoring data. SpO₂ values therefore represent documented pulse oximetry readings rather than predefined clinical targets. Comparable exposure definitions derived from discrete electronic health record oxygen saturation values have been used in prior large observational cohorts (10, 11). All diagnostic, procedure, medication and laboratory codes used for cohort construction and covariate adjustment are outlined in Supplementary Table 2.

### Patient selection

Adult patients (≥18 years) with a stroke diagnosis who subsequently required invasive mechanical ventilation within 48 h of stroke onset were identified. The index event was defined as the patient’s first stroke diagnosis accompanied by invasive mechanical ventilation and a documented SpO₂ reading within 48 h. All SpO₂ results recorded within 48 h of the index stroke diagnosis, ascertained by pulse oximetry (LOINC code 59408-5), were retrieved and used to determine whether a patient met the inclusion and exclusion criteria. Patients were required to have at least one such value. Patients were classified as restrictive if all recorded values fell between 92 and 96% inclusive, and as liberal if all recorded values were ≥97%. These thresholds correspond to the conservative and liberal oxygenation ranges employed in randomized trials of oxygen targets in critically ill adults (15, 16, 26).

Patients were excluded if any recorded value fell below 92%, or if recorded values fell into both ranges. Requiring all values to fall within a single range prevents assignment of a single exposure category to patients whose documented oxygenation spanned both, and the two cohorts are therefore non-overlapping by construction. The 48-h window was selected to capture oxygenation during the early phase of stroke, when cerebral autoregulation is impaired and tissue at risk remains susceptible to secondary injury; fixed early exposure windows of this kind have been used to define oxygenation exposure in prior critically ill and brain-injured cohorts (10, 11). While these criteria reduced the available sample size, they were considered essential to preserve the internal validity of the exposure classification. After applying all inclusion and exclusion criteria, 17,207 patients comprised the eligible study cohort.

Two pre-specified subgroup analyses were conducted: one restricted to patients with AIS and one restricted to patients with ICH. Each subgroup cohort was constructed independently within the TriNetX platform using separate queries. For the AIS-only cohort, a diagnosis of ICH was included as an exclusion criterion, and conversely, a diagnosis of AIS was excluded from the ICH-only cohort, ensuring complete mutual exclusivity between subtype populations. Because each cohort was built and matched independently, the subgroup sample sizes are not arithmetically derived from the primary cohort and should not be expected to sum to the primary cohort total. This is a recognized methodological feature of independent query construction within federated database platforms such as TriNetX.

### Variables

Matching is performed only among patients with documented values for all covariates included in the propensity model. Covariates included in the propensity score model were selected *a priori* based on clinical relevance and included: age at index event (continuous), sex, race and ethnicity, body mass index (BMI, continuous), Glasgow Coma Scale score ≤9 (dichotomous), and the following comorbidities: hypertensive diseases, ischemic heart disease, chronic ischemic heart disease, heart failure, atrial fibrillation and flutter, cardiomyopathy, chronic lower respiratory diseases, chronic obstructive pulmonary disease, asthma, nicotine dependence, pneumonia (unspecified organism), bacterial pneumonia, viral pneumonia, pleural effusion, shock, personal history of transient ischemic attack or cerebral infarction, pneumothorax, and personal history of other respiratory diseases. Propensity score matching was performed using a complete-case approach. Patients with missing values for any covariate included in the matching model were excluded from the matched analysis. No imputation of missing data was performed.

### Outcomes

The primary outcome was all-cause mortality, evaluated at 30 days and 90 days following the index event, defined as a patient’s first stroke diagnosis accompanied by invasive mechanical ventilation and a documented SpO₂ reading within 48 h falling within pre-defined ranges. Secondary outcomes included tracheostomy placement within 30 days of the index event and the development of VAP during the hospitalization.

### Statistical analysis

Continuous variables are presented as mean ± standard deviation (SD), and categorical variables are reported as counts and percentages. Baseline characteristics between oxygenation groups were compared using standardized mean differences (SMD). Propensity score matching was performed separately for the AIS, ICH, and combined AIS + ICH cohorts using 1:1 nearest-neighbor matching without replacement. Patients in the restrictive oxygenation cohort were matched to those in the liberal cohort using a greedy nearest-neighbor algorithm to minimize absolute differences in propensity scores. Standardized mean differences (SMD) < 0.10 was considered indicative of adequate balance after matching. Matching was conducted independently within each cohort; therefore, matched sample sizes differed across analyses. The combined AIS + ICH cohort was constructed *de novo* rather than as the sum of the subtype cohorts, and propensity matching was repeated within this population.

The primary outcome of all-cause mortality was analyzed using time-to-event methods. Kaplan–Meier survival curves were generated, and differences between groups were assessed using the log-rank test. Hazard ratios (HR) with 95% confidence intervals (CIs) were estimated using Cox proportional hazards regression models. Secondary outcomes were analyzed using risk ratios (RR) and odds ratios (OR). All statistical tests were two-sided, and a *p*-value <0.05 was considered statistically significant. Analyses were performed within the TriNetX database, and only aggregate results were exported.

## Results

### Baseline characteristics of the cohort

A total of 17,207 mechanically ventilated stroke patients met inclusion criteria, of whom 2,366 received restrictive oxygen saturation targets and 14,841 received liberal oxygenation prior to PSM. For subgroup analyses, the AIS cohort comprised 1,214 and 7,463 patients in the restrictive and liberal groups prior to matching (1,200 per group after PSM), and the ICH cohort comprised 743 and 6,891 patients (728 per group after PSM) (Figure 1). Baseline characteristics of the AIS and ICH subgroups before and after matching are presented in Supplementary Tables 3 and 4.

**Figure 1:**
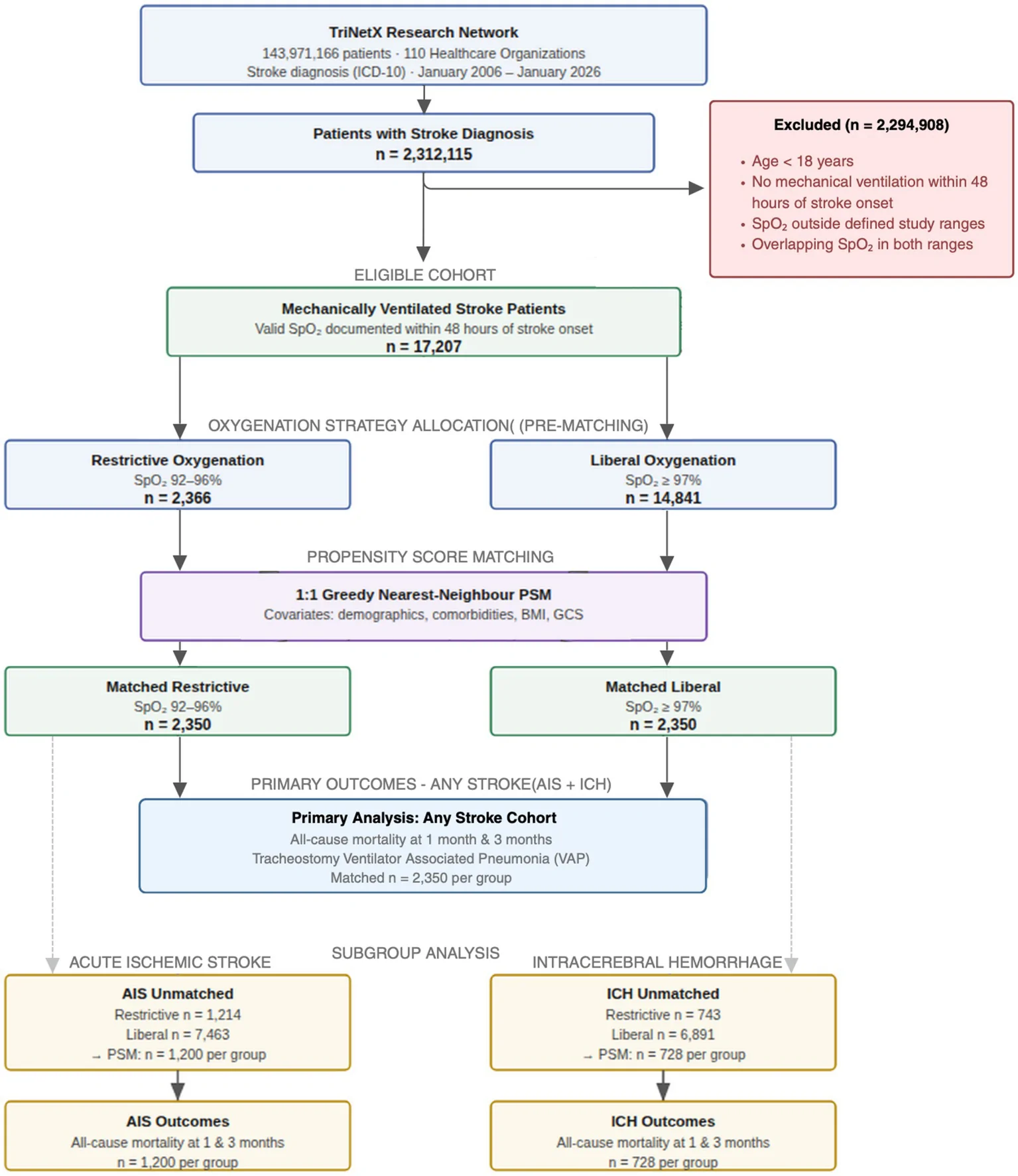
Study flow diagram. Flow diagram illustrating patient selection and cohort assembly. A total of 2,312,115 patients with a stroke diagnosis were identified within the TriNetX Research Network between January 2006 and January 2026. Following application of inclusion and exclusion criteria, 17,207 mechanically ventilated stroke patients with valid SpO₂ documentation within 48 h of stroke onset comprised the eligible cohort. Patients were stratified into restrictive (SpO₂ 92–96%; *n* = 2,366) and liberal (SpO₂ ≥ 97%; *n* = 14,841) oxygenation groups, yielding 2,350 matched patients per group following 1:1 propensity score matching. Subgroup analyses were conducted independently for acute ischemic stroke (AIS; *n* = 1,200 per group after matching) and intracerebral hemorrhage (ICH; *n* = 728 per group after matching). AIS, acute ischemic stroke; ICH, intracerebral hemorrhage; PSM, propensity score matching; SpO₂, peripheral oxygen saturation; VAP, ventilator-associated pneumonia.

Baseline characteristics of the restrictive and liberal oxygenation cohorts before and after propensity score matching are presented in Table 1. Prior to matching, several demographic and clinical variables demonstrated imbalance between groups, including age, race distribution, and selected cardiopulmonary comorbidities. The largest standardized mean differences were observed for race and body mass index. After 1:1 nearest-neighbor propensity score matching without replacement, 2,350 patients remained in each cohort. All baseline covariates achieved adequate balance, with standardized mean differences <0.1 across matched variables (Table 1).

**Table 1:** Baseline characteristics of mechanically ventilated stroke patients receiving restrictive or liberal oxygen saturation targets.

Characteristic	Before Matching		*P*-value	Std. Diff.	After Matching		*P*-value	Std. Diff.
	Restrictive (*n* = 2,366)	Liberal (*n* = 14,841)			Restrictive (*n* = 2,350)	Liberal (*n* = 2,350)		
Demographics								
Age at Index, mean ± SD	62.2 ± 19	59.1 ± 20.3	<0.0001	**0.1559**	62.1 ± 19	62.6 ± 19	0.3717	0.0261
Male	1,435 (60.68%)	8,103 (54.62%)	<0.0001	**0.1228**	1,422 (60.511%)	1,415 (60.21%)	0.8346	0.0061
White	1,655 (69.98%)	8,361 (56.36%)	<0.0001	**0.2852**	1,646 (70.043%)	1,606 (68.34%)	0.2063	0.0369
Hispanic or Latino	367 (15.52%)	1,674 (11.28%)	<0.0001	**0.1245**	367 (15.617%)	378 (16.09%)	0.6604	0.0128
Black or African American	219 (9.26%)	3,200 (21.57%)	<0.0001	**0.346**	219 (9.319%)	225 (9.57%)	0.7648	0.0087
American Indian or Alaska Native	91 (3.85%)	201 (1.355%)	<0.0001	**0.1571**	85 (3.617%)	90 (3.83%)	0.7001	0.0112
Asian	52 (2.20%)	790 (5.325%)	<0.0001	**0.1649**	52 (2.213%)	51 (2.17%)	0.9206	0.0029
Comorbidities								
Hypertensive diseases	1,095 (46.3%)	6,265 (42.23%)	0.0002	0.082	1,081 (46%)	1,041 (44.3%)	0.241	0.0342
Ischemic heart diseases	553 (23.38%)	2,878 (19.4%)	<0.0001	0.0972	546 (23.234%)	533 (22.71%)	0.6521	0.0132
Chronic ischemic heart disease	457 (19.32%)	2,472 (16.66%)	0.0014	0.0693	454 (19.319%)	441 (18.77%)	0.6291	0.0141
Chronic lower respiratory diseases	439 (18.56%)	2,177 (14.68%)	<0.0001	**0.1046**	432 (18.383%)	404 (17.19%)	0.2855	0.0312
Heart failure	437 (18.48%)	2,255 (15.20%)	<0.0001	0.0877	430 (18.298%)	413 (17.57%)	0.5181	0.0189
Nicotine dependence	381 (16.11%)	1,798 (12.12%)	<0.0001	**0.1148**	370 (15.745%)	367 (15.62%)	0.9042	0.0035
Atrial fibrillation and flutter	362 (15.31%)	2,015 (13.583%)	0.0241	0.0491	358 (15.234%)	345 (14.69%)	0.595	0.0155
Pneumonia, unspecified organism	351 (14.84%)	1,669 (11.25%)	<0.0001	**0.1068**	344 (14.638%)	332 (14.13%)	0.6179	0.0146
Chronic obstructive pulmonary disease	298 (12.6%)	1,207 (8.136%)	<0.0001	**0.1468**	291 (12.383%)	273 (11.617%)	0.4191	0.0236
Pleural effusion	259 (10.951%)	1,320 (8.898%)	0.0013	0.0687	255 (10.851%)	254 (10.809%)	0.9626	0.0014
History of TIA or cerebral infarction	197 (8.33%)	1,266 (8.534%)	0.7411	0.0073	195 (8.298%)	175 (7.447%)	0.2787	0.0316
Shock	170 (7.188%)	891 (6.006%)	0.0265	0.0476	168 (7.149%)	145 (6.17%)	0.1784	0.0393
Asthma	141 (5.962%)	908 (6.121%)	0.7645	0.0067	140 (5.957%)	137 (5.83%)	0.8526	0.0054
Cardiomyopathy	133 (5.624%)	851 (5.736%)	0.8264	0.0049	132 (5.617%)	131 (5.574%)	0.9494	0.0019
Bacterial pneumonia	112 (4.736%)	579 (3.903%)	0.0554	0.041	110 (4.681%)	100 (4.26%)	0.4802	0.0206
Viral pneumonia	74 (3.129%)	224 (1.51%)	<0.0001	**0.1077**	69 (2.936%)	70 (2.98%)	0.9314	0.0025
Labs								
BMI (mean ± SD)	30 ± 8.08	27.6 ± 7.26	<0.0001	**0.3131**	30 ± 8.1	29.5 ± 7.38	0.1226	0.0641
Glasgow Coma Scale ≤9	124 (5.243%)	481 (3.24%)	<0.0001	0.0994	117 (4.979%)	108 (4.60%)	0.5386	0.0179

Baseline characteristics of patients receiving restrictive versus liberal oxygen saturation before and after propensity score matching. Before matching, the restrictive cohort included 2,366 patients and the liberal cohort included 14,841 patients. After 1:1 propensity score matching, both cohorts contained 2,350 patients. Data are presented as *n* (%) for categorical variables and mean ± SD for continuous variables. SMD <0.1 indicates negligible imbalance. Counts for categorical variables may not sum to cohort totals due to variable-level missingness within the TriNetX network. Percentages were calculated using available-case denominators for each variable. Bold values indicate standardized differences ≥0.1 before matching, denoting meaningful covariate imbalance.

### Primary outcome

After PSM, the restrictive oxygen group was associated with significantly higher all-cause mortality compared with liberal oxygen group at both 1 and 3 months of follow-up. At 1 month, the restrictive oxygen group was associated with an increased hazard of death (HR 1.13, 95% CI 1.03–1.23; *p* = 0.008), and this association persisted at 3 months (HR 1.11, 95% CI 1.02–1.20; *p* = 0.014) (Figure 2).

**Figure 2:**
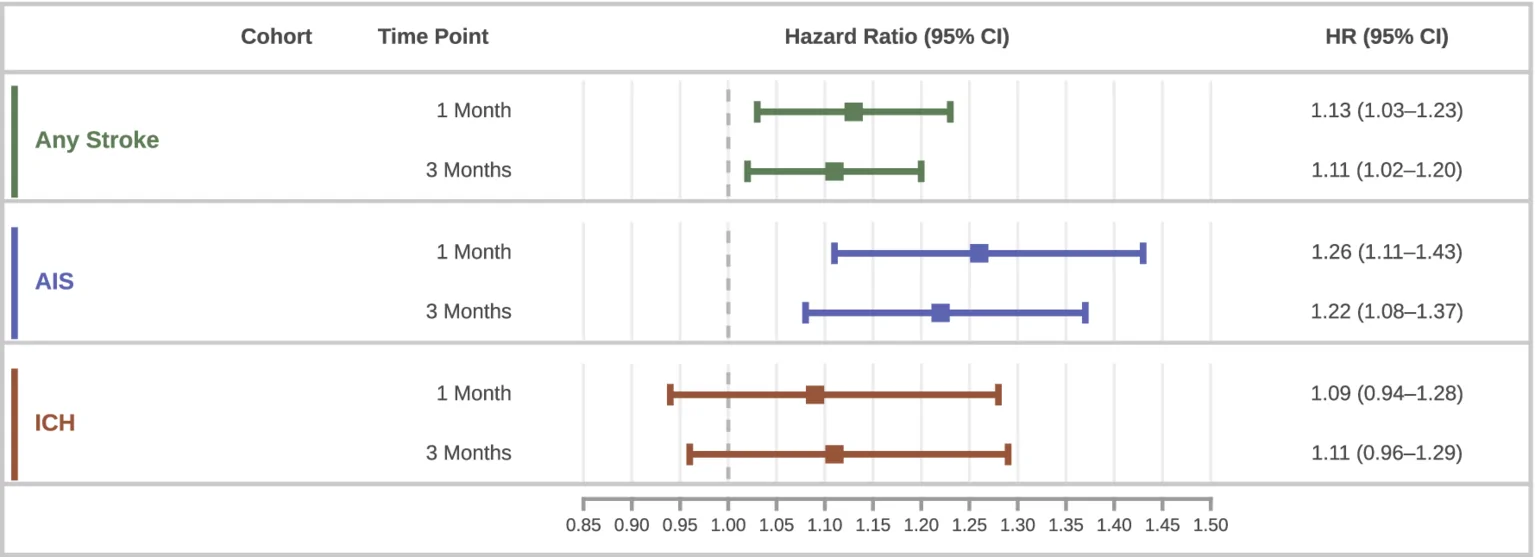
Forest plot for all-cause mortality (reference: liberal). Forest plot displaying hazard ratios (HR) with 95% confidence intervals for all-cause mortality at 1 and 3 months of follow-up in propensity-score-matched cohorts. Three stroke populations are shown: Any Stroke (combined AIS + ICH), Acute Ischemic Stroke (AIS), and Intracerebral Hemorrhage (ICH). The dashed vertical reference line at HR = 1.0 denotes no difference between strategies; values to the right indicate higher mortality risk associated with restrictive oxygenation. All cohorts were propensity-score-matched on baseline demographic and clinical characteristics.

In the AIS subgroup, restrictive oxygenation was similarly associated with higher mortality at 1 month (HR 1.26, 95% CI 1.11–1.43) and at 3 months (HR 1.22, 95% CI 1.08–1.37) (Figure 2).

In contrast, in the ICH subgroup, restrictive oxygenation was not associated with a statistically significant difference in mortality at either time point (1 month HR 1.09, 95% CI 0.94–1.28; 3 months HR 1.11, 95% CI 0.96–1.29) (Figure 2).

Kaplan–Meier survival analyses demonstrated separation of the survival curves between restrictive and liberal oxygenation in the combined stroke cohort and in the AIS subgroup, whereas the curves did not separate in the ICH subgroup (Figure 3).

**Figure 3:**
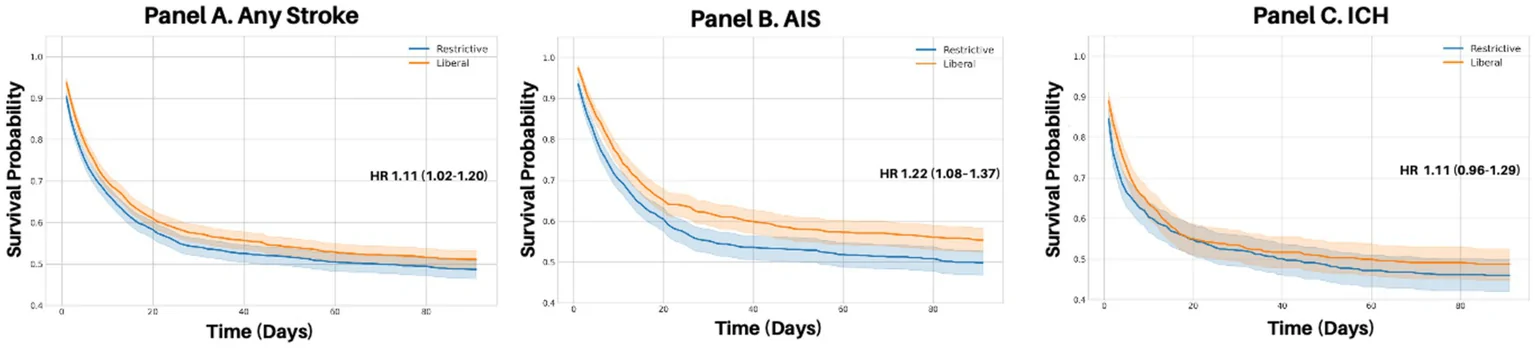
Kaplan–Meier survival curves for all-cause mortality comparing restrictive versus liberal oxygen saturation targets (reference: liberal). Panel **(A)** shows the combined cohort of AIS and ICH. Panel **(B)** shows patients with acute ischemic stroke. Panel **(C)** shows patients with intracerebral hemorrhage. Survival probability is displayed over 90 days following index stroke. The shaded regions represent 95% confidence intervals. Differences between groups were assessed using the log-rank test.

### Secondary outcomes

After PSM, restrictive oxygenation was associated with a significantly lower rate of tracheostomy at 30 days compared with liberal oxygenation (8.5% vs. 13.1%) and lower risk (RR 0.653, 95% CI 0.552–0.773, *p* < 0.001) (Table 2). There was no statistically significant difference in VAP between restrictive and liberal oxygenation groups (0.6% vs. 0.89%). The relative risk was 0.667 (95% CI 0.339–1.311, *p* = 0.238) (Table 2). The low number of events did not allow subgroup analyses by stroke subtype of secondary outcomes.

**Table 2:** Secondary outcomes comparing restrictive and liberal oxygen saturation targets at 30 days.

Outcome	Restrictive events	Liberal events	Risk difference % (95% CI)	Risk ratio (95% CI)	Odds ratio (95% CI)	*P*-Value
Tracheostomy	201/2,350 (8.55%)	308/2,350 (13.1%)	−4.55% (−6.31 – −2.8%)	0.653 (0.55–0.77)	0.620 (0.515–0.75)	<0.0001
Ventilator-associated pneumonia	14/2,350 (0.60%)	21/2,350 (0.89%)	−0.30% (−0.79 – +0.19%)	0.667 (0.34–1.31)	0.665 (0.337–1.31)	0.2381

Comparison of secondary clinical outcomes between restrictive and liberal oxygen saturation targets in the propensity score-matched cohort (*n* = 2,350 per group). Data are presented as the number of events with percentages. Risk differences are expressed as absolute percentage differences with 95% confidence intervals (CI). Risk ratios (RR) with 95% CIs quantify the relative effect of restrictive compared with liberal oxygenation. Negative risk differences indicate fewer events in the restrictive group.

## Discussion

In this large, multicenter, retrospective PSM study of stroke patients, oxygenation profiles during the first 48 h of mechanical ventilation in stroke patients was associated with variable short-term outcomes. Restrictive oxygen saturation during this early period was associated with significantly higher 1- and 3-month mortality in stroke, and specifically in patients with AIS; whereas no significant mortality difference was observed in ICH. These findings suggest that early oxygenation strategy during the initial phase of stroke may have important implications in this patient population. While these associations do not imply causation due to the observational design of this analysis, they do suggest a correlation.

The first 48 h represent a particularly critical window in acute stroke (6, 13, 14, 17). During this period, cerebral autoregulation may be impaired, and hypoperfused but not ischemic brain tissue (penumbra) remains highly susceptible to secondary injury (17, 18). Even mild changes in oxygen delivery could theoretically exacerbate ongoing ischemia (13, 14, 17). The association between restrictive oxygenation during early ventilation and increased mortality in AIS observed in our study supports this hypothesis. Conversely, concerns regarding oxygen-related toxicity, including oxidative stress and cerebral vasoconstriction associated with hyperoxia, have led to increasing adoption of more conservative oxygen strategies in general ICU populations (4, 5, 8, 10, 15, 18–20). However, randomized trials evaluating conservative versus liberal oxygenation in heterogeneous critically ill cohorts may not be directly generalizable to mechanically ventilated stroke patients, whose cerebral perfusion dysregulation and pathophysiology differ substantially (8, 10, 17–19). Our findings suggest that extrapolation of restrictive oxygen paradigms from general ICU populations to acute ischemic stroke may warrant caution, particularly during the early ventilation period.

In contrast, we did not observe a statistically significant association between early restrictive oxygenation and mortality in ICH. The pathophysiologic drivers of outcome in ICH differ from those in AIS and include hematoma expansion, mass effect, and perihematomal edema rather than primary perfusion failure (8). It is possible that early oxygen saturation targets have less influence on outcome than the primary hemorrhagic cerebral injury. Alternatively, differences in statistical power or heterogeneity in ICH severity may have masked detectable associations. Notably, although not statistically significant, the hazard ratios in ICH trended in the same direction as AIS, constituting a signal favoring liberal oxygenation strategies (9, 11). The non-significant association may reflect limited statistical power due to smaller sample size.

Our findings should be considered against the subgroup analyses of the randomized oxygenation trials, which provide the closest available evidence in brain-injured populations. Two post-hoc analyses of the ICU-ROX trial are pertinent. Among participants with suspected hypoxic-ischaemic encephalopathy, conservative oxygen therapy was associated with lower 180-day mortality (21). Among participants with acute brain pathologies other than hypoxic-ischaemic encephalopathy, a group comprising ischaemic stroke, subarachnoid hemorrhage and traumatic brain injury, 180-day mortality was numerically higher in those allocated to conservative oxygen than to liberal oxygen (34.5% versus 26.9%; absolute difference 7.6 percentage points, 95% CI −4.7 to 19.9), although this comparison did not reach statistical significance; the interaction between the two neurological subgroups was significant (*p* = 0.02) (22). This apparent heterogeneity was considered of sufficient plausibility to be formalized as a prespecified hypothesis in Mega-ROX Brains, which is randomizing patients with non-hypoxic-ischaemic acute brain injury to conservative or liberal oxygenation (23).

The direction of association observed in our cohort is consistent with that reported in the non-hypoxic-ischaemic brain injury subgroup, and differs principally from the pooled estimates derived from general intensive care populations (24). Several considerations may account for this. The randomized trials enrolled predominantly non-neurological patients, in whom tissue at risk of secondary ischaemic injury is unlikely to represent the principal determinant of outcome, and acute stroke constituted a small proportion of the enrolled populations. The contrast in oxygenation examined in the present study is narrower than that tested in several of the randomized comparisons. Most importantly, our exposure was observed rather than allocated, and residual confounding by unmeasured factors cannot be excluded. These findings are accordingly interpreted as hypothesis-generating and concordant with the question presently under prospective investigation, rather than as evidence supporting a change in clinical practice.

An additional finding of interest was the lower 30-day tracheostomy rate observed in the restrictive oxygen group despite higher mortality. This pattern may reflect survivorship bias. However, reasons for intubation, trajectory of neurologic recovery, and goals-of-care discussions may also contribute to this finding (19). Those factors could not be measured in our study and represent an important limitation. Importantly, no significant difference was observed in ventilator-associated pneumonia, suggesting that differences in early oxygenation strategies do not affect infectious complications. The low rate of VAP may be explained by the inclusion of patients who remained on mechanical ventilation for less than 48 h, hence without the potential to develop this complication.

Taken together, these results suggest that oxygenation strategy during the first 48 h of mechanical ventilation may represent a modifiable factor associated with short-term outcome in stroke, specifically in AIS (9, 11, 19), representing a potential area for prospective research.

Prospective studies are needed to determine optimal saturation thresholds. Randomized trials focusing on disease-specific oxygen targets, rather than broad ICU approaches, may be particularly important to clarify whether early liberal oxygenation confers benefit in AIS or whether individualized titration strategies can balance the competing risks of hypoxemia and hyperoxia (6, 7, 15, 20, 25).

### Limitations

This study has several important limitations. First, its retrospective observational design precludes causal inference. Although propensity score matching was performed separately within the AIS, ICH and AIS + ICH combined cohorts to minimize confounding, residual confounding by unmeasured variables remains possible. Stroke severity measures such as infarct volume, hematoma size, imaging findings, and detailed neurologic examination data were not available within the federated electronic health record network and could not be directly incorporated into matching. Second, exposure classification required every recorded SpO₂ value to fall within a single range, a deliberately conservative definition that eliminates misclassification from patients whose documented oxygenation crossed both categories. This yields a cleanly separated exposure contrast at the cost of a smaller analytic sample weighted toward patients with fewer documented saturations. The thresholds used to define the exposure groups are also adjacent, and the resulting contrast in oxygenation between cohorts is correspondingly narrow; a wider separation would provide a more distinct comparison, and the magnitude of any association observed across adjacent categories should be interpreted with this in mind. Oxygen exposure was further defined based on recorded oxygen saturation rather than continuous arterial oxygen tension measurements. Detailed data regarding FiO₂ (Fraction of Inspired Oxygen) levels and PaO₂ thresholds were not available. Therefore, the analysis reflects associations with oxygenation strategy rather than precise quantification of hyperoxemia or hypoxemia. Third, propensity score matching was conducted independently within each cohort, and matching was re-run prior to outcome analysis. As a result, matched sample sizes differed across analyses, and hazard ratios for combined and subtype cohorts were derived from separately matched populations rather than a single hierarchical model. Fourth, secondary outcomes were restricted to coding captured within the network. The low number of VAP events limited statistical power and precluded subtype-specific analyses. Additionally, we were unable to directly account for withdrawal-of-life-sustaining therapy decisions, which may influence both mortality and tracheostomy rates. Finally, follow-up was limited to 3 months; thus, these findings cannot be extrapolated to longer-term outcomes.

## Conclusion

In this study of stroke patients, restrictive oxygen saturation in the first 48 h of mechanical ventilation was associated with significantly higher short-term mortality in the general stroke population and in patients with acute ischemic stroke, but not in those with intracerebral hemorrhage. This study suggests that oxygenation strategy may have subtype-specific implications in stroke patients. Our findings underscore the importance of disease-specific oxygenation research rather than general ICU oxygenation protocols. Prospective randomized trials are needed to determine optimal oxygen saturation targets and to clarify the balance between preventing hypoxemia and avoiding potential oxygen-related toxicity in this high-risk population.

## Data Availability

The data analyzed in this study were obtained from the TriNetX Research Network and are subject to the following licenses/restrictions: the data are de-identified and available only to TriNetX network members under a data use agreement. Requests to access these datasets should be directed to TriNetX, LLC (https://trinetx.com).
