# Supplementary material for: Association of liberal versus restrictive oxygen saturation targets with mortality in mechanically ventilated stroke patients: a multicenter retrospective propensity-matched analysis
Source: Front Neurol. 2026 Oct 19;17:1840183. doi: 10.3389/fneur.2026.1840183 (PMC13724415; doi:10.3389/fneur.2026.1840183)
Supplement: Supplementary file 1 [file Table_1.docx]

**Supplementary Table 1.** STROBE Checklist for Cohort Studies

| **Section** | **Item** | **Recommendation** | **Reported in** |
| --- | --- | --- | --- |
| **Title and abstract** | 1a | Indicate the study design with a commonly used term in the title or abstract | Title; Abstract |
|  | 1b | Provide an informative and balanced summary of what was done and what was found | Abstract |
| **Introduction** | 2 | Explain the scientific background and rationale for the investigation | Introduction |
|  | 3 | State specific objectives, including any prespecified hypotheses | Introduction (final paragraph) |
| **Methods** | 4 | Present key elements of study design early in the paper | Methods: Study design and data source |
|  | 5 | Describe the setting, locations, and relevant dates, including periods of recruitment, exposure, follow-up, and data collection | Methods: Study design and data source; Patient selection; Outcomes; Figure 1 |
|  | 6a | Give the eligibility criteria and the sources and methods of selection of participants; describe methods of follow-up | Methods: Patient selection; Outcomes |
|  | 6b | For matched studies, give matching criteria and number of exposed and unexposed | Methods: Variables; Statistical analysis; Results: Baseline characteristics of the cohort; Figure 1 |
|  | 7 | Clearly define all outcomes, exposures, predictors, potential confounders, and effect modifiers | Methods: Patient selection; Variables; Outcomes |
|  | 8 | For each variable of interest, give sources of data and details of methods of assessment | Methods: Study design and data source; Supplementary Table 2 |
|  | 9 | Describe any efforts to address potential sources of bias | Methods: Patient selection; Statistical analysis; Discussion: Limitations |
|  | 10 | Explain how the study size was arrived at | Methods: Patient selection; Results: Baseline characteristics of the cohort; Figure 1 |
|  | 11 | Explain how quantitative variables were handled in the analyses | Methods: Patient selection; Variables |
|  | 12a | Describe all statistical methods, including those used to control for confounding | Methods: Statistical analysis |
|  | 12b | Describe any methods used to examine subgroups and interactions | Methods: Patient selection; Statistical analysis |
|  | 12c | Explain how missing data were addressed | Methods: Variables |
|  | 12d | If applicable, explain how loss to follow-up was addressed | Methods: Statistical analysis (time-to-event analysis) |
|  | 12e | Describe any sensitivity analyses | Not performed |
| **Results** | 13a | Report numbers of individuals at each stage of the study | Results: Baseline characteristics of the cohort; Figure 1 |
|  | 13b | Give reasons for non-participation at each stage | Methods: Patient selection; Figure 1 |
|  | 13c | Consider use of a flow diagram | Figure 1 |
|  | 14a | Give characteristics of study participants and information on exposures and potential confounders | Results: Baseline characteristics of the cohort; Table 1; Supplementary Tables 3 and 4 |
|  | 14b | Indicate number of participants with missing data for each variable of interest | Table 1 footnote; Supplementary Tables 3 and 4 |
|  | 14c | Summarise follow-up time | Methods: Outcomes; Figure 3 |
|  | 15 | Report numbers of outcome events or summary measures over time | Results: Primary outcome; Secondary outcomes; Table 2; Figure 3 |
|  | 16a | Give unadjusted estimates and, if applicable, confounder-adjusted estimates and their precision; make clear which confounders were adjusted for | Methods: Variables; Results: Primary outcome; Secondary outcomes; Table 2; Figure 2 |
|  | 16b | Report category boundaries when continuous variables were categorized | Methods: Patient selection; Variables |
|  | 16c | If relevant, consider translating estimates of relative risk into absolute risk | Results: Secondary outcomes; Table 2 |
|  | 17 | Report other analyses done, such as analyses of subgroups and interactions | Results: Primary outcome; Figure 2; Figure 3 |
| **Discussion** | 18 | Summarise key results with reference to study objectives | Discussion (first paragraph) |
|  | 19 | Discuss limitations of the study, including sources of potential bias or imprecision | Discussion: Limitations |
|  | 20 | Give a cautious overall interpretation of results considering objectives, limitations, and other relevant evidence | Discussion; Conclusion |
|  | 21 | Discuss the generalisability (external validity) of the study results | Discussion; Limitations |
| **Other information** | 22 | Give the source of funding and the role of the funders | Funding statement |

*Checklist of items reported according to the Strengthening the Reporting of Observational Studies in Epidemiology (STROBE) statement for cohort studies. Recommendations are abridged. Locations refer to sections, tables, and figures of the article.*
