# Supplementary material for: Association of liberal versus restrictive oxygen saturation targets with mortality in mechanically ventilated stroke patients: a multicenter retrospective propensity-matched analysis
Source: Front Neurol. 2026 Oct 19;17:1840183. doi: 10.3389/fneur.2026.1840183 (PMC13724415; doi:10.3389/fneur.2026.1840183)
Supplement: Supplementary file 2 [file Table_2.docx]

# Supplementary Table 2.

Diagnostic, Procedure, Medication, and Laboratory Codes Used for Cohort Construction and Covariate Adjustment

| Domain | Variable / Definition | Code System | Codes Used | Purpose in Study |
| --- | --- | --- | --- | --- |
|  | Acute Ischemic Stroke (AIS) | ICD-10-CM | I63.x | Index diagnosis |
|  | Intracerebral Hemorrhage (ICH) | ICD-10-CM | I61.x | Index diagnosis |
|  | Prior TIA / Prior Cerebral Infarction | ICD-10-CM | G45.x; Z86.73 | Baseline comorbidity |
| Exposure Variable | Pulse Oximetry (SpO₂) | LOINC | 59408-5 | Exposure definition (92–96% restrictive; ≥97% liberal) |
| Mechanical Ventilation | Invasive Mechanical Ventilation | ICD-10-PCS | 5A1935Z; 5A1945Z; 5A1955Z | Inclusion criterion (≤48h of stroke) |
| Demographics | Age, Sex, Race, Ethnicity | Native TriNetX fields | — | Matching covariates |
| Cardiovascular | Hypertensive diseases | ICD-10-CM | I10–I16 | PSM covariate |
|  | Ischemic heart disease | ICD-10-CM | I20–I25 | PSM covariate |
|  | Chronic ischemic heart disease | ICD-10-CM | I25.x | PSM covariate |
|  | Heart failure | ICD-10-CM | I50.x | PSM covariate |
|  | Cardiomyopathy | ICD-10-CM | I42.x | PSM covariate |
|  | Atrial fibrillation/flutter | ICD-10-CM | I48.x | PSM covariate |
|  | Other cardiac arrhythmias | ICD-10-CM | I49.x | PSM covariate |
|  | Aortic aneurysm/dissection | ICD-10-CM | I71.x | PSM covariate |
| Pulmonary | Chronic lower respiratory diseases | ICD-10-CM | J40–J47 | PSM covariate |
|  | COPD | ICD-10-CM | J44.x | PSM covariate |
|  | Asthma | ICD-10-CM | J45.x | PSM covariate |
|  | Pneumonia, unspecified | ICD-10-CM | J18.9 | PSM covariate |
|  | Bacterial pneumonia | ICD-10-CM | J13–J15 | PSM covariate |
|  | Viral pneumonia | ICD-10-CM | J12.x | PSM covariate |
|  | Pleural effusion | ICD-10-CM | J90 | PSM covariate |
|  | Pneumothorax | ICD-10-CM | J93.x | PSM covariate |
| Infectious | Other sepsis | ICD-10-CM | A41.x | PSM covariate |
| Neurologic / Severity | Glasgow Coma Scale | ICD-10-CM | R40.2x | Stroke severity proxy |
|  | Cerebral edema | ICD-10-CM | G93.6 | Severity covariate |
|  | Brain compression | ICD-10-CM | G93.5 | Severity covariate |
|  | Shock | ICD-10-CM | R57.x | Severity covariate |
| Substance Use | Nicotine dependence | ICD-10-CM | F17.x | PSM covariate |
| Laboratory / Anthropometric | Body Mass Index (BMI) | ICD-10-CM | Z68.x | PSM covariate |
|  |  | Native measurement | Recorded BMI value | Continuous covariate |
| Secondary Outcomes | Tracheostomy | ICD-10-PCS | 0B110F4, 0B113F4, 0B113Z4, 0B114F4, 0B114Z4 | 30-day outcome |
|  | Ventilator-associated pneumonia | ICD-10-CM | J95.851 | 30-day outcome |

All diagnoses were identified using ICD-10-CM codes. Procedures were identified using ICD-10-PCS and/or CPT codes where available within the TriNetX Research Network. Laboratory measurements were identified using LOINC codes. Code ranges reflect hierarchical grouping within TriNetX ontology expansion. Exposure definitions required pulse oximetry documentation within 48 hours of index stroke diagnosis.
