# Supplementary material for: Association of liberal versus restrictive oxygen saturation targets with mortality in mechanically ventilated stroke patients: a multicenter retrospective propensity-matched analysis
Source: Front Neurol. 2026 Oct 19;17:1840183. doi: 10.3389/fneur.2026.1840183 (PMC13724415; doi:10.3389/fneur.2026.1840183)
Supplement: Supplementary file 3 [file Table_3.docx]

**Supplementary Table 3.** Baseline Characteristics of Acute Ischemic Stroke Patients Receiving Restrictive vs Liberal Oxygen Saturation Targets

|  | **Before Matching** | |  |  | **After Matching** | |  |  |
| --- | --- | --- | --- | --- | --- | --- | --- | --- |
| **Characteristic** | **Restrictive (n=1,214)** | **Liberal (n=7,463)** | **P-value** | **SMD** | **Restrictive (n=1,200)** | **Liberal**  **(n=1,200)** | **P-value** | **SMD** |
| **Demographics** |  |  |  |  |  |  |  |  |
| Age, years (mean ± SD) | 64.3 ± 16.7 | 62.1 ± 19.0 | 0.0001 | **0.123** | 64.3 ± 16.7 | 64.0 ± 17.0 | 0.618 | 0.0204 |
| Male | 730 (60.13%) | 4,034 (54.05%) | <0.0001 | **0.123** | 718 (59.83%) | 721 (60.08%) | 0.9005 | 0.0051 |
| White | 856 (70.51%) | 3,947 (52.89%) | <0.0001 | **0.3686** | 843 (70.25%) | 815 (67.92%) | 0.2162 | 0.0505 |
| Black or African American | 123 (10.13%) | 1,958 (26.24%) | <0.0001 | **0.4269** | 123 (10.25%) | 117 (9.75%) | 0.6831 | 0.0167 |
| Hispanic or Latino | 158 (13.02%) | 764 (10.24%) | 0.0036 | 0.0867 | 155 (12.92%) | 146 (12.17%) | 0.5791 | 0.0226 |
| Asian | 28 (2.31%) | 319 (4.27%) | 0.0012 | **0.1105** | 28 (2.33%) | 34 (2.83%) | 0.4401 | 0.0315 |
| American Indian or Alaska Native | 31 (2.55%) | 112 (1.50%) | 0.0075 | 0.0748 | 30 (2.50%) | 41 (3.42%) | 0.1851 | 0.0541 |
| **Comorbidities** |  |  |  |  |  |  |  |  |
| Hypertensive diseases | 612 (50.41%) | 3,849 (51.57%) | 0.4523 | 0.0233 | 600 (50.00%) | 562 (46.83%) | 0.1206 | 0.0634 |
| Ischemic heart diseases | 338 (27.84%) | 2,052 (27.50%) | 0.8023 | 0.0077 | 329 (27.42%) | 314 (26.17%) | 0.4893 | 0.0282 |
| Chronic ischemic heart disease | 266 (21.91%) | 1,770 (23.72%) | 0.1685 | 0.043 | 262 (21.83%) | 249 (20.75%) | 0.5168 | 0.0265 |
| Nicotine dependence | 221 (18.20%) | 1,052 (14.10%) | 0.0002 | **0.1118** | 211 (17.58%) | 200 (16.67%) | 0.5512 | 0.0243 |
| Chronic lower respiratory diseases | 276 (22.74%) | 1,434 (19.21%) | 0.0042 | 0.0865 | 267 (22.25%) | 255 (21.25%) | 0.5527 | 0.0242 |
| Heart failure | 283 (23.31%) | 1,718 (23.02%) | 0.8233 | 0.0069 | 277 (23.08%) | 274 (22.83%) | 0.8842 | 0.0059 |
| Atrial fibrillation and flutter | 217 (17.88%) | 1,418 (19.00%) | 0.3523 | 0.029 | 216 (18.00%) | 217 (18.08%) | 0.9577 | 0.0022 |
| Pneumonia, unspecified organism | 221 (18.20%) | 1,166 (15.62%) | 0.0229 | 0.0689 | 214 (17.83%) | 207 (17.25%) | 0.7071 | 0.0153 |
| Pleural effusion | 162 (13.34%) | 899 (12.05%) | 0.2004 | 0.039 | 158 (13.17%) | 150 (12.50%) | 0.6254 | 0.0199 |
| COPD | 197 (16.23%) | 870 (11.66%) | <0.0001 | **0.1322** | 189 (15.75%) | 180 (15.00%) | 0.6105 | 0.0208 |
| Shock | 116 (9.56%) | 655 (8.78%) | 0.3766 | 0.027 | 111 (9.25%) | 115 (9.58%) | 0.7798 | 0.0114 |
| Asthma | 85 (7.00%) | 552 (7.40%) | 0.6247 | 0.0153 | 83 (6.92%) | 75 (6.25%) | 0.5102 | 0.0269 |
| Cardiomyopathy | 89 (7.33%) | 630 (8.44%) | 0.193 | 0.0412 | 85 (7.08%) | 97 (8.08%) | 0.3548 | 0.0378 |
| Bacterial pneumonia | 73 (6.01%) | 399 (5.35%) | 0.3421 | 0.0288 | 71 (5.92%) | 70 (5.83%) | 0.9308 | 0.0035 |
| Pneumothorax/air leak | 26 (2.14%) | 163 (2.18%) | 0.9252 | 0.0029 | 26 (2.17%) | 24 (2.00%) | 0.775 | 0.0117 |
| Viral pneumonia | 45 (3.71%) | 157 (2.10%) | 0.0006 | 0.0956 | 40 (3.33%) | 43 (3.58%) | 0.7375 | 0.0137 |
| Personal history of TIA/cerebral infarction | 129 (10.63%) | 909 (12.18%) | 0.1218 | 0.0489 | 127 (10.58%) | 131 (10.92%) | 0.7921 | 0.0108 |
| Personal history of other respiratory diseases | 18 (1.48%) | 84 (1.13%) | 0.2843 | 0.0315 | 16 (1.33%) | 14 (1.17%) | 0.7133 | 0.015 |
| **Labs** |  |  |  |  |  |  |  |  |
| BMI, kg/m² (mean ± SD) | 30.2 ± 8.05 | 27.9 ± 7.35 | <0.0001 | **0.2984** | 30.2 ± 8.05 | 30.1 ± 7.96 | 0.9398 | 0.0042 |
| Glasgow Coma Scale ≤9 | 46 (3.79%) | 285 (3.82%) | 0.96 | 0.0016 | 43 (3.58%) | 42 (3.50%) | 0.9121 | 0.0045 |

*Baseline characteristics of ischemic stroke patients receiving restrictive versus liberal oxygen saturation targets before and after 1:1 propensity score matching. Before matching, cohorts included 1,214 restrictive and 7,463 liberal patients; after matching, 1,200 patients remained in each cohort. Standardized mean differences (SMD) ≥0.1 before matching are shown in bold and indicate baseline imbalance; all covariates achieved adequate balance after matching (SMD <0.1). Counts for categorical variables may not sum to cohort totals due to variable-level missingness within the TriNetX network. Percentages were calculated using available-case denominators for each variable.*
