# Supplementary material for: Association of liberal versus restrictive oxygen saturation targets with mortality in mechanically ventilated stroke patients: a multicenter retrospective propensity-matched analysis
Source: Front Neurol. 2026 Oct 19;17:1840183. doi: 10.3389/fneur.2026.1840183 (PMC13724415; doi:10.3389/fneur.2026.1840183)
Supplement: Supplementary file 4 [file Table_4.docx]

**Supplementary Table 4.** Baseline Characteristics of Intracerebral Hemorrhage Patients Receiving Restrictive vs Liberal Oxygen Saturation Targets

|  | **Before Matching** | |  |  | **After Matching** | |  |  |
| --- | --- | --- | --- | --- | --- | --- | --- | --- |
| **Characteristic** | **Restrictive**  **(n=743)** | **Liberal (n=6,891)** | **P-value** | **SMD** | **Restrictive**  **(n=728)** | **Liberal**  **(n=728)** | **P-value** | **SMD** |
| **Demographics** |  |  |  |  |  |  |  |  |
| Age, years (mean ± SD) | 57.00 ± 23.00 | 54.70 ± 22.10 | 0.0073 | **0.102** | 57.10 ± 23.10 | 57.30 ± 22.60 | 0.8508 | 0.01 |
| Male | 457 (61.51%) | 3,850 (55.87%) | 0.0032 | **0.1147** | 443 (60.85%) | 453 (62.23%) | 0.5901 | 0.03 |
| White | 501 (67.43%) | 3,840 (55.72%) | <0.0001 | **0.2424** | 495 (68.00%) | 473 (64.97%) | 0.2219 | 0.06 |
| Hispanic or Latino | 154 (20.73%) | 944 (13.70%) | <0.0001 | **0.187** | 151 (20.74%) | 144 (19.78%) | 0.6481 | 0.02 |
| Black or African American | 63 (8.48%) | 1,255 (18.21%) | <0.0001 | **0.2892** | 63 (8.65%) | 66 (9.07%) | 0.782 | 0.01 |
| American Indian or Alaska Native | 37 (4.98%) | 121 (1.76%) | <0.0001 | **0.1794** | 30 (4.12%) | 32 (4.40%) | 0.7952 | 0.01 |
| Asian | 19 (2.56%) | 428 (6.21%) | <0.0001 | **0.1792** | 19 (2.61%) | 21 (2.89%) | 0.7485 | 0.02 |
| **Comorbidities** |  |  |  |  |  |  |  |  |
| Hypertensive diseases | 252 (33.92%) | 2,035 (29.53%) | 0.0132 | 0.0943 | 243 (33.38%) | 232 (31.87%) | 0.5386 | 0.03 |
| Ischemic heart diseases | 106 (14.27%) | 708 (10.27%) | 0.0008 | **0.1219** | 101 (13.87%) | 109 (14.97%) | 0.5507 | 0.03 |
| Chronic ischemic heart disease | 92 (12.38%) | 608 (8.82%) | 0.0014 | **0.1158** | 88 (12.09%) | 98 (13.46%) | 0.4324 | 0.04 |
| Nicotine dependence | 85 (11.44%) | 669 (9.71%) | 0.1328 | 0.0563 | 82 (11.26%) | 75 (10.30%) | 0.5542 | 0.03 |
| Chronic lower respiratory diseases | 77 (10.36%) | 632 (9.17%) | 0.2875 | 0.0402 | 75 (10.30%) | 75 (10.30%) | 1.00 | <0.0001 |
| Heart failure | 78 (10.50%) | 497 (7.21%) | 0.0013 | **0.1158** | 74 (10.17%) | 88 (12.09%) | 0.2433 | 0.06 |
| Pneumonia, unspecified organism | 76 (10.23%) | 489 (7.10%) | 0.0019 | **0.1115** | 73 (10.03%) | 72 (9.89%) | 0.9303 | <0.01 |
| Atrial fibrillation and flutter | 67 (9.02%) | 472 (6.85%) | 0.0284 | 0.0803 | 64 (8.79%) | 72 (9.89%) | 0.4712 | 0.04 |
| Pleural effusion | 53 (7.13%) | 423 (6.14%) | 0.2867 | 0.04 | 50 (6.87%) | 66 (9.07%) | 0.1215 | 0.08 |
| Chronic obstructive pulmonary disease | 43 (5.79%) | 288 (4.18%) | 0.0409 | 0.0739 | 42 (5.77%) | 45 (6.18%) | 0.7401 | 0.02 |
| Shock | 35 (4.71%) | 228 (3.31%) | 0.0465 | 0.0715 | 31 (4.26%) | 34 (4.67%) | 0.7034 | 0.02 |
| Asthma | 29 (3.90%) | 305 (4.43%) | 0.5079 | 0.0262 | 28 (3.85%) | 27 (3.71%) | 0.8907 | 0.01 |
| Cardiomyopathy | 22 (2.96%) | 180 (2.61%) | 0.5735 | 0.0212 | 22 (3.02%) | 31 (4.26%) | 0.2079 | 0.07 |
| Bacterial pneumonia | 24 (3.23%) | 164 (2.38%) | 0.1554 | 0.0515 | 22 (3.02%) | 22 (3.02%) | 1.00 | <0.0001 |
| Viral pneumonia | 17 (2.29%) | 61 (0.89%) | 0.0003 | **0.1124** | 15 (2.06%) | 14 (1.92%) | 0.8512 | 0.01 |
| Personal history of TIA/cerebral infarction | 14 (1.88%) | 164 (2.38%) | 0.395 | 0.0343 | 14 (1.92%) | 10 (1.37%) | 0.4103 | 0.04 |
| Pneumothorax | 15 (2.02%) | 99 (1.44%) | 0.2138 | 0.0447 | 14 (1.92%) | 17 (2.34%) | 0.586 | 0.03 |
| Personal history of other respiratory diseases | 10 (1.35%) | 45 (0.65%) | 0.0339 | 0.0697 | 10 (1.37%) | 10 (1.37%) | 1.00 | <0.0001 |
| **Labs** |  |  |  |  |  |  |  |  |
| BMI, kg/m² (mean ± SD) | 29.10 ± 8.28 | 26.70 ± 6.96 | <0.0001 | **0.32** | 29.00 ± 8.38 | 28.70 ± 7.45 | 0.7376 | 0.03 |
| GCS ≤9 | 36 (4.85%) | 172 (2.50%) | 0.0003 | **0.13** | 29 (3.98%) | 34 (4.67%) | 0.5196 | 0.03 |

*Baseline characteristics of mechanically ventilated intracerebral hemorrhage patients receiving restrictive versus liberal oxygenation before and after propensity score matching. Before matching, the restrictive cohort included 743 patients and the liberal cohort included 6,891 patients. After 1:1 propensity score matching, both cohorts contained 728 patients. Standardized mean differences (SMD) ≥0.1 before matching are shown in bold and indicate baseline imbalance. Race/ethnicity categories are not mutually exclusive; patients may have more than one race code documented in the TriNetX database, therefore percentages do not sum to 100%. Similarly, sex categories also do not sum to 100% due to Unknown gender entries in the TriNetX database. Counts for categorical variables may not sum to cohort totals due to variable-level missingness within the TriNetX network. Percentages were calculated using available-case denominators for each variable. The reduction in sample size reflects exclusion of unmatched patients to achieve covariate balance between groups. Data are presented as n (%) for categorical variables and mean ± SD for continuous variables.*
